# Two New Coumarins from *Talaromyces flavus*

**DOI:** 10.3390/molecules191220880

**Published:** 2014-12-12

**Authors:** Jun-Wei He, Da-Peng Qin, Hao Gao, Run-Qiao Kuang, Yang Yu, Xing-Zhong Liu, Xin-Sheng Yao

**Affiliations:** 1 Institute of Traditional Chinese Medicine and Natural Products, College of Pharmacy, Jinan University, Guangzhou 510632, China; 2 Research Center of Natural Resources of Chinese Medicinal Materials and Ethnic Medicine, Jiangxi University of Traditional Chinese Medicine, Nanchang 330004, China; 3 State Key Laboratory of Mycology, Institute of Microbiology, Chinese Academy of Sciences, Beijing 100190, China

**Keywords:** *Talaromyces flavus*, coumarin, structure elucidation, biological activities

## Abstract

Two new coumarins, talacoumarins A (**1**) and B (**2**), were isolated from the ethyl acetate extract of the wetland soil-derived fungus *Talaromyces flavus* BYD07-13. Their structures were elucidated by spectroscopic data (NMR, MS) analyses. The absolute configuration of C-12 in **1** was assigned using the modified Mosher’s method, whereas that of C-12 in **2** was deduced via the circular dichroism data of its corresponding [Rh_2_(OCOCF_3_)_4_] complex. Compounds **1** and **2** were evaluated for their anti-Aβ_42_ aggregation, cytotoxic, and antimicrobial activities. The results showed that the two compounds had moderate anti-Aβ_42_ aggregation activity, and this is the first report on the Aβ_42_ inhibitory aggregation activity of coumarins.

## 1. Introduction

**Figure 1:**
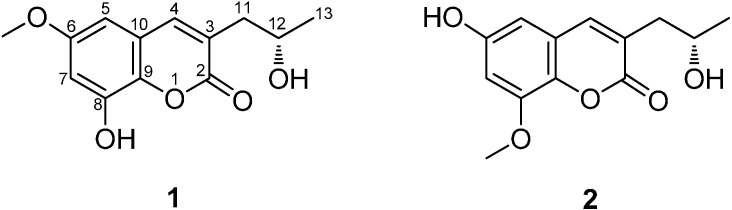
Chemical structures of compounds **1** and **2**.

Fungi of the genus *Talaromyces* have been reported to produce a series of bioactive compounds [1,2,3,4,5]. Metabolites produced by *T. flavus* have also been reviewed [5]. In our searching for bioactive secondary metabolites from fungi, we have previously isolated a series of polyesters [6], as well as one sequiterpene [7] from the wetland derived fungus *Talaromyces flavus* BYD07-13, which was collected from a soil sample in Baiyangdian (Hebei Province, China). Ongoing chemical study on this fungus has now resulted in the isolation and identification of two new coumarins, named talacoumarins A (**1**) and B (**2**) (Figure 1). In this paper, we describe the isolation, structure elucidation, as well as the anti-Aβ_42_ aggregation, cytotoxic, and antimicrobial activities of **1** and **2**.

## 2. Results and Discussion

Compound **1** was isolated as a yellow amorphous powder, and its molecular formula was determined as C_13_H_14_O_5_ on the basis of the HR-ESI-MS (*m/z* 273.0740, calcd. 273.0739 [M+Na]^+^). The UV spectrum showed an absorption band with λ_max_ 208, 257, and 294 nm, characteristic of coumarins [8]. The IR spectrum of **1** displayed absorption bands for hydroxyl (3261 cm^−1^), carbonyl (1684 cm^−1^) and aromatic (1592 and 1497 cm^−1^) groups. The ^1^H-NMR spectrum (Table 1) of **1** exhibited a pair of *meta*-positioned aromatic protons at δ_H_ 6.63 and 6.61 (each, 1H, d, *J* = 2.7 Hz), an olefinic proton at δ_H_ 7.72 (1H, s). It also displayed the signals of one methoxyl at δ_H_ 3.74 (3H, s), one methyl at δ_H_ 1.10 (3H, d, *J* = 6.2 Hz), an oxygenated methyne proton at δ_H_ 3.92 (1H, m), one methylene protons at δ_H_ 2.54 (1H, dd, *J* = 13.5, 4.9 Hz), 2.45 (1H, dd, *J* = 13.5, 7.8 Hz), as well as a phenolic hydroxyl at δ_H_ 10.20 (1H, s), and an alcoholic hydroxyl at δ_H_ 4.62 (1H, d, *J* = 4.5 Hz). The ^13^C-NMR spectrum (Table 1) combined with DEPT 135 spectrum displayed 13 resonances for an ester carbonyl carbon (δ_C_ 161.0), eight aromatic/olefinic carbons (δ_C_ 155.6, 145.1, 141.1, 136.4, 126.5, 120.2, 104.9, 100.1), one oxymethine carbon (δ_C_ 64.2), one methoxyl group (δ_C_ 55.4), one methylene carbon (δ_C_ 40.3), and one methyl group (δ_C_ 23.4).

**Figure 2:**
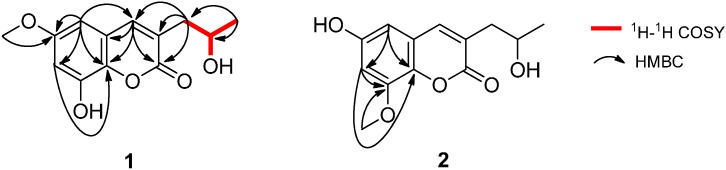
Selected ^1^H-^1^H COSY and HMBC correlations of **1** and **2**.

The ^1^H-^1^H COSY correlations between H-12 and H_a,b_-11/H_3_-13/12-OH, in combination with the HMBC correlations from H_a,b_-11 to C-2/C-3/C-4, H-4 to C-2/C-9/C-10, H-5 to C-4/C-6/C-7/C-9, H-7 to C-9, and H_3_-13 to C-11/C-12, indicating the presence of 3-propyl-6,8-dioxy coumarin moiety in **1** (Figure 2). Moreover, the methoxyl group was located at C-6 by the HMBC correlation from 6-OCH_3_ to C-6. Considering the ^13^C-NMR chemical shifts of C-8 (δ_C_ 145.1) and C-12 (δ_C_ 64.2), as well as molecular formula, the two carbons should be connected with hydroxyl groups. Thus, the structure of **1** was fully elucidated to be as indicated in Figure 1, and it was named talacoumarin A.

**Table 1:** ^13^C- (100 MHz) and ^1^H- (400 MHz) NMR data for compounds **1** and **2** in DMSO-*d*_6_ (δ in ppm).

Position	1		2	
	δ_C_	δ_H_ (*J* in Hz)	δ_C_	δ_H_ (*J* in Hz)
1				
2	161.0		160.9	
3	126.5		126.6	
4	141.1	7.72 (s)	140.9	7.69 (s)
5	100.1	6.63 (d, 2.7)	102.8	6.51 (d, 2.3)
6	155.6		153.9	
7	104.9	6.61 (d, 2.7)	102.4	6.66 (d, 2.3)
8	145.1		147.0	
9	136.4		135.7	
10	120.2		119.9	
11	40.3	2.45 (dd, 13.5, 7.8), H_a_	40.2	2.44 (dd, 13.6, 7.7), H_a_
		2.54 (dd, 13.5, 4.9), H_b_		2.52 (overlapped), H_b_
12	64.2	3.92 (m)	64.2	3.92 (m)
13	23.4	1.10 (d, 6.2)	23.4	1.09 (d, 6.1)
8-OH		10.20 (s)		
12-OH		4.62 (d, 4.5)		
6-OCH_3_	55.4	3.74 (s)		
8-OCH_3_			55.9	3.84 (s)

**Figure 3:**
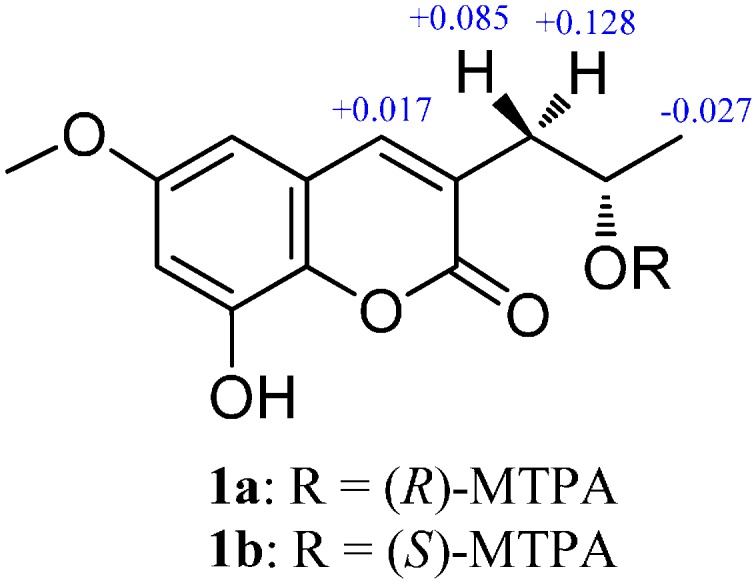
Δδ values (in ppm) = δ_S_ − δ_R_ obtained for (*S*)-(**1a**) and (*R*)-MTPA (**1b**) esters.

The absolute configuration at C-12 was determined to be *S* by comparison of its optical rotation value with that of (*S*)-orthosporin (**1**: ${\left[\text{α}\right]}_{\text{D}}^{20}$ +56.0 (*c* 0.5, CH_3_OH); (*S*)-orthosporin: ${\left[\text{α}\right]}_{\text{D}}^{22}$ +61.8 (*c* 1.0, CH_3_OH) [9]). As confirmation, the absolute configuration at C-12 was established by the modified Mosher’s method [10]. The values of the (*R*)- and (*S*)-MTPA esters **1a** and **1b** also indicated the *S* configuration for C-12 (Figure 3).

Compound **2** possessed the same molecular formula and UV absorption characteristic as that of **1**, suggesting **2** may be a coumarin isomer of **1**. The NMR spectroscopic data (Table 1) suggested **2** was very similar to **1**, except for the position of the methoxy group, which was located at C-8 for **2** instead of at C-6 based on the HMBC correlations from 8-OCH_3_ to C-8, from H-5 to C-7/C-9, and from H-7 to C-8/C-9 (Figure 2). With the aid of the ^1^H-^1^H COSY, HSQC, and HMBC correlations, the planar structure of **2** was established and all the ^1^H- and ^13^C-NMR signals were assigned.

**Figure 4:**
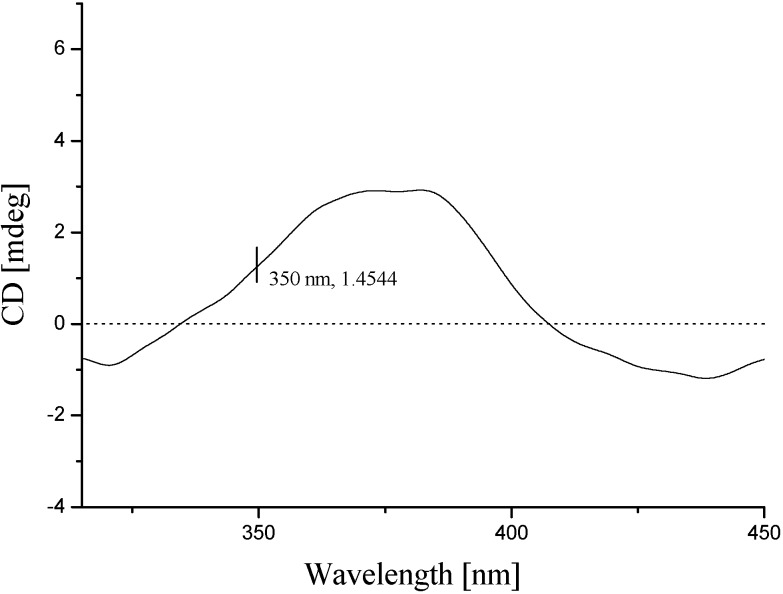
The CD spectrum of the Rh complex of **2** with the inherent CD spectrum subtracted.

The optical rotation of **2** (${\left[\text{α}\right]}_{\text{D}}^{20}$ +55.6 (*c* 0.5, CH_3_OH)) was consistent with that of **1**, which suggested that **2** had the same configuration. The absolute configuration at C-12 was determined on the basis of the circular dichroism of the complex formed *in situ* with [Rh_2_(OCOCF_3_)_4_] [11,12], with the inherent contribution of the ligand subtracted. Upon addition of [Rh_2_(OCOCF_3_)_4_] to a solution of **2** in CH_2_Cl_2_, a metal complex was formed, acting as an auxiliary chromophore. It has been demonstrated that the sign of the E band (at *ca.* 350 nm) can be used to correlate the absolute configuration of a secondary and tertiary alcohol by applying the bulkiness rule. In this case, the Rh complex of **2** displayed a positive E band (Figure 4), correlating with a 12*S* absolute configuration. Hence, the structure of **2** was established as shown in Figure 1 and named to be talacoumarin B.

So far, natural products from fungi with the 3-alkyl-6,8-dioxycoumarin scaffold are relatively rare, and only eight such compounds have been reported [8,13,14,15]. The inhibitory activities against Aβ_42_ aggregation of compounds **1** and **2**, along with that of the crude extract, were tested by a thioflavin T (ThT) assay [16] with epigallocatechin gallate (EGCG) as the positive control. Compounds **1** and **2** showed moderate anti-Aβ_42_ aggregation activities, with relative inhibitory rates of (49.33 ± 3.16)% and (44.99 ± 3.64)% [the positive control EGCG had a relative inhibitory rate of (67.23 ± 2.51)%] at the concentration of 100 μM, while the crude extract has no activity. This represents the first report of the anti-Aβ_42_ aggregation activity of coumarins. Compounds **1**, **2**, and the crude extract were also evaluated for the cytotoxicity against five human tumor cell lines (HL-60, SMMC-7721, A-549, MCF-7, and SW480) and the antimicrobial activity against *Escherichia coil*, *Staphylococcus aureus*, *Candida albicans*, and *Aspergillus niger*. However, none of them showed any cytotoxic (IC_50_ > 40 μM) or antimicrobial activities (MIC > 1.0 mg/mL).

## 3. Experimental Section

### 3.1. General Procedures

Optical rotations were measured using a JASCO P-1020 polarimeter (JASCO Corporation, Tokyo, Japan). The IR spectra (KBr) were recorded on a JASCO FT/IR-480 plus Fourier transform infrared spectrometer (JASCO Corporation). The UV spectra were recorded in CH_3_OH using a JASCO V-550 UV/Vis spectrophotometer (JASCO Corporation). ^1^H- (400 MHz), ^13^C- (100 MHz), DEPT 135 (100 MHz), and 2D (^1^H-^1^H COSY, HSQC, and HMBC) NMR spectra were recorded in DMSO-*d*_6_ on a Bruker AV 400 spectrometer using solvent signals (DMSO-*d*_6_: δ_H_ 2.50/δ_C_ 39.5) as an internal standard (Bruker Corporation, Fallanden, Switzerland). HR-ESI-MS were measured on a Waters Synapt G2 TOF mass spectrometer (Waters Corporation, Manchester, UK). Column chromatographies (CCs) were carried out on silica gel (200–300 mesh, Marine Chemical Group Corporation, Qingdao, China), and ODS (60–80 µm, YMC, Tokyo, Japan). Silica gel GF_254_ (Marine Chemical Group Corporation) was used for analytical TLC. The analytical HPLC was performed on a Shimadzu HPLC system equipped with a LC-20AB pump, and a SPD-20A diode array detector (Shimadzu, Kyoto, Japan), using a Phenomenex Gemini C18 column (5 μm, 4.6 mm × 250 mm, Phenomenex Inc., Torrance, CA, USA). The Preparative HPLC was performed on Shimadzu LC-6AD system equipped with a LC-6AD pump, and a SPD-M20A detector (Shimadzu), using an RP-18 column (5 μm, 21.2 mm × 250 mm, Gemini, Phenomenex Inc.; detector set at 220 and 254 nm).

### 3.2. Fungal Material and Culture

The strain of *Talaromyces flavus* (No.BYD07-13) was identified on the basis of the morphological characters and gene sequence analyses. The ITS, beta-tubulin, and calmodulin sequences of the strain have been deposited at GenBank as KF917583, KF917584, and KF917585, respectively. It was deposited in the culture collection at the Institute of Traditional Chinese Medicine and Natural Products, College of Pharmacy, Jinan University, Guangzhou, China. The fermentation of No.BYD07-13 was described in our previous paper [6].

### 3.3. Extraction and Isolation

The fermented material was extracted three times with EtOAc (3 × 6.0 L), and the organic solvent was evaporated to dryness under vacuum to afford the crude extract (46.2 g), which was dissolved in 90% v/v aqueous CH_3_OH (500 mL) and partitioned against an equal volume of cyclohexane. The aqueous CH_3_OH layer was evaporated to dryness under reduced pressure to give an aqueous CH_3_OH extract (w, 28.1 g), which was subjected to ODS column chromatography (CC) using a CH_3_OH-H_2_O gradient elution (30:70, 50:50, 70:30, and 100:0, v/v) to give four fractions (w1 to w4). The fraction w1 (574 mg) was further separated by ODS CC using a CH_3_OH/H_2_O gradient elution (30:70, 40:60, and 50:50, v/v, each 0.5 L) to give three subfractions (w1a to w1c). The subfraction w1a (351 mg) was purified by preparative HPLC eluting with CH_3_CN/H_2_O (v/v 15:85, flow rate: 8 mL/min) to afford compounds **1** (37.9 mg, t_R_ 21.2 min) and **2** (5.0 mg, t_R_ 16.1 min).

### 3.4. Spectroscopic Data

*Talacoumarin A* (**1**). Yellow amorphous powder, ${\left[\text{α}\right]}_{\text{D}}^{20}$ +56.0 (*c* 0.5, CH_3_OH); UV (CH_3_OH) λ_max_ (log *ε*) 208 (4.65), 257 (4.07), 294 (4.27) nm; IR (KBr): ν_max_ 3261, 2962, 2920, 2853, 1684, 1592, 1510, 1454, 1392, 1351, 1203, 1164, 1109, 1053, 855, 840 cm^−1^; ^1^H- (DMSO-*d*_6_) and ^13^C-NMR (DMSO-*d*_6_) data, see Table 1. HR-ESI-MS: *m/z* 273.0740 ([M+Na]^+^, C_13_H_14_O_5_Na; calcd. 273.0739).

*Talacoumarin B* (**2**). Yellow amorphous powder, ${\left[\text{α}\right]}_{\text{D}}^{20}$ +55.6 (*c* 0.5, CH_3_OH); UV (CH_3_OH) λ_max_ (log *ε*) 208 (4.65), 257 (4.07), 294 (4.27) nm; IR (KBr): ν_max_ 3261, 2962, 2920, 2853, 1684, 1592, 1510, 1454, 1392, 1351, 1292, 1203, 1109, 1053, 855, 841 cm^−1^; ^1^H-NMR (DMSO-*d*_6_) and ^13^C-NMR (DMSO-*d*_6_) data, see Table 1. HR-ESI-MS: *m/z* 273.0737 ([M+Na]^+^, C_13_H_14_O_5_Na; calcd. 273.0739).

### 3.5. Preparation of (S)- and (R)-MTPA Esters of **1** (**1a** and **1b**)

A solution of **1** (0.5 mg) in pyridine-*d*_5_ (0.5 mL) was treated with (*S*)-MTPA chloride (8 µL) under an atmosphere of nitrogen in an NMR tube. The mixture was stirred at room temperature for 20 h to obtain the (*R*)-MTPA ester (**1b**). The same procedure was used to prepare the (*S*)-MTPA ester (**1a**) with (*R*)-MTPA chloride.

### 3.6. Absolute Configuration of the Secondary Alcohol in **2**

According to a published procedure [11,12], **2** (0.5 mg) was dissolved in a dry solution of the stock [Rh_2_(OCOCF_3_)_4_] complex (1.0 mg) in CH_2_Cl_2_ (200 μL). The first CD spectrum was recorded immediately after mixing, and its time evolution was monitored until stationary (about 10 min after mixing). The inherent CD was subtracted. The observed sign of the E band at *ca.* 350 nm in the induced CD spectrum was correlated to the absolute configuration of the C-12 secondary alcohol moiety.

### 3.7. ThT Fluorescence Assay

The ThT fluorescene assay was performed as described in our previous paper [16], with EGCG as the positive control. Biological activity was determined as relative inhibitory activity (V_i_) for each sample according to the formula: V_i_ = 100 − [(F_i_ − F_b_)/F_0_] × 100, where F_i_ is the fluorescence value of the sample, F_b_ is its bank value, and F_0_ is the fluorescence value for free aggregation of a sample of Aβ_42_ incubated in the same buffer/HFIP/DMSO system and in absence of inhibitors.

### 3.8. Cytotoxicity Assay

Cytotoxic activity was tested against five human cell lines (HL-60, SMMC-7721, A-549, MCF-7, and SW480) using the MTT method as described in our previous paper [6]. Cisplatin and paclitaxel were used as the positive controls.

### 3.9. Antimicrobial Assay

The antimicrobial activity against *E. coil*, *S. aureus*, *C. albicans*, and *A. niger* were evaluated by an agar dilution method, which described in our previous paper [7]. Tobramycin was used as the positive control.

## 4. Conclusions

Our investigation on the metabolites of the wetland soil-derived fungus *T. flavus* BYD07-13 resulted in the isolation of two new coumarins, named talacoumarins A (**1**) and B (**2**). Compounds **1** and **2** both have the 3-alkyl-6,8-dioxycoumarin moiety, which is relatively rare in fungal metabolites. The absolute configurations of **1** and **2** were determined by the modified Mosher’s method and the CD analysis of the *in situ* formed [Rh_2_(OCOCF_3_)_4_] complex. It is noteworthy that the absolute configuration of branched chain alcohol of 3-alkyl-6,8-dioxycoumarin was determined for the first time. Compounds **1** and **2** showed moderate anti-Aβ_42_ aggregation activity, making this the first report on the Aβ_42_ inhibitory aggregation activity of coumarins.
